# Double lung, unlike single lung transplantation might provide a protective effect on mortality and bronchiolitis obliterans syndrome

**DOI:** 10.1186/s13019-017-0666-5

**Published:** 2017-11-25

**Authors:** Mohammed Fakhro, Ellen Broberg, Lars Algotsson, Lennart Hansson, Bansi Koul, Ronny Gustafsson, Per Wierup, Richard Ingemansson, Sandra Lindstedt

**Affiliations:** 1 Department of Cardiothoracic Surgery, Skåne University Hospital, Lund University, Lund, Sweden; 2 Department of Thoracic Intensive Care and Anesthesia, Skåne University Hospital, Lund University, Lund, Sweden; 3 Department of Pulmonary Medicine, Skåne University Hospital, Lund University, Lund, Sweden

**Keywords:** Lung transplantation, Bronchiolitis obliterans, Graft rejection, Graft survival, Survival rate

## Abstract

**Background:**

Survival after lung transplantation (LTx) is often limited by bronchiolitis obliterans syndrome (BOS).

**Method:**

Survey of 278 recipients who underwent LTx. The endpoint used was BOS (BOS grade ≥ 2), death or Re-lung transplantation (Re-LTx) assessed by competing risk regression analyses.

**Results:**

The incidence of BOS grade ≥ 2 among double LTx (DLTx) recipients was 16 ± 3% at 5 years, 30 ± 4% at 10 years, and 37 ± 5% at 20 years, compared to single LTx (SLTx) recipients whose corresponding incidence of BOS grade ≥ 2 was 11 ± 3%, 20 ± 4%, and 24 ± 5% at 5, 10, and 20 years, respectively (*p* > 0. 05). The incidence of BOS grade ≥ 2 by major indications ranked in descending order: other, PF, CF, COPD, PH and AAT1 (*p* < 0. 05). The mortality rate by major indication ranked in descending order: COPD, PH, AAT1, PF, Other and CF (*p* < 0. 05).

**Conclusion:**

No differences were seen in the incidence of BOS grade ≥ 2 regarding type of transplant, however, DLTx recipients showed a better chance of survival despite developing BOS compared to SLTx recipients. The highest incidence of BOS was seen among CF, PF, COPD, PH, and AAT1 recipients in descending order, however, CF and PF recipients showed a better chance of survival despite developing BOS compared to COPD, PH, and AAT1 recipients.

## Background

Chronic lung allograft dysfunction (CLAD) remains the major barrier to long-term success after lung transplantation [1–3]. The primary cause of death after LTx is CLAD. The development of CLAD is rare in the first year after LTx, but the rate increases quickly with cumulative incidence reported to be as high as 40% to 80% within the first five years [4–7]. CLAD that manifests early after transplantation reportedly shows a poorer prognosis than late-onset CLAD. Bronchiolitis obliterans (BO) is the pathologic pattern of injury most commonly seen in lung transplant recipients with progressive loss of lung function. It is believed to be due to chronic allograft rejection and is characterized by the obliteration of small airways by fibromyxoid granulation tissue. Distribution is patchy and difficult to detect with transbronchial biopsy [3, 7]. Because BO is difficult to document histologically, the International Society for Heart and Lung Transplantation (ISHLT) in 1993 established criteria for its physiologic counterpart, bronchiolitis obliterans syndrome (BOS). This diagnosis requires a permanent 20% drop in the forced expiratory volume in 1 s (FEV1) not attributable to a concurrent process [8]. Since 2014 the diagnosis for chronic rejection was further broadened to CLAD, which includes restrictive allograft syndrome (RAS). CLAD can also be diagnosed by CT scan with visual signs of small airway disease and lung biopsies with severe narrowing or complete obstruction of the small airways [8].

In Sweden two centers perform lung transplantation for a population of about 10 million, all of whom are covered by national health insurance. Skåne University Hospital is one of these centers. This retrospective report reviews the 25-year experience of Skåne University Hospital, Lund University Lung Transplant Program with particular emphasis on chronic rejection between different subgroups of recipients and type of transplant procedure performed. Because of the retrospective nature of this study, chronic rejection is referred to as BOS rather than the recently termed CLAD.

## Patients and method

Between January 1990 to June 2014, 278 patients underwent lung transplantation at Skåne University Hospital, Lund University. Double lung transplantation (DLTx) was performed in 172 patients, single lung transplantation (SLTx) in 97 patients, and HLTx in 9 patients. Of these, 129 were male and 149 were female. Re-lung transplantation (Re-LTx) was performed in 15 patients. Among the Re-LTx recipients, of whom 5 were female and 10 were male, 7 recipients had a DLTx and 8 had a SLTx.

In the present study the median age was 51 years with a range of 12–71 years. The major indications were defined as chronic obstructive pulmonary disease (COPD) (*n* = 67), cystic fibrosis (CF) (*n* = 54), α1-antitrypsin deficiency (AAT1) (*n* = 55), pulmonary fibrosis (PF) (*n* = 38), pulmonary hypertension (PH) (*n* = 39), and a group deemed as “other” (*n* = 25), which included bronchiectasis, sarcoidosis, bronchioalveolary cancer, silicosis, and graft-vs-host disease (GVHD).

HLTx was performed via median sternotomy in 7 patients and via a clamshell (bilateral anterolateral thoracotomy with a transverse sternotomy) incision in the 4th intercostal space in 2 patients. SLTx and DLTx were performed in standard fashion. SLTx was performed through a posterolateral thoracotomy in 86 patients, via clamshell in 7 patients, and via median sternotomy in 4 patients. DLTx was performed through a clamshell-incision in 146 patients, via median sternotomy in 17 patients, and via anterolateral thoracotomy in 9 patients.

Preoperative respiratory support was used in 13 operations (CF 4, PF 5, Re-LTx 3, PH 1). Preoperative ECMO (extracorporeal membrane oxygenation) support was used in 12 operations (CF 6, PF 3, ARDS 1, PH 1, Re-LTx 1).

Intraoperative circulatory support in the form of extracorporeal circulation (ECC) was used in 105 cases, and intraoperative ECMO was used in 73 cases. Intraoperative circulatory support was not used in 115 cases. Recipient characteristics are shown in Table 1.

**Table 1:** Recipient characteristics

Baseline Characteristics of the 278 Patients	
Variable	Median (Range) or No. (%)
Recipient age, year	51 (12–71)
Recipient primary disease	
Cystic fibrosis	54
Pulmonary fibrosis	38
Chronic obstructive pulmonary disease (COPD)	67
α1-antitrypsin deficiency (AAT1)	55
Pulmonary hypertension (PH)	39
Other	25
Transplant type	
DLTx	172
SLTx	97
HLTx	9
Re-LTx	15
DLTx	7
SLTx	8
Gender	
Female	149
Male	129
Transplant year	
1990–2002	126
2003–2014	167
Preoperative ventilator	13
Preoperative ECMO	12
Perioperative ECC	105
Perioperative ECMO	73
Postoperative ECMO	30

### Chronic allograft dysfunction

According to ISHLT guidelines, BOS is defined as more than 20% decline in FEV_1_ from the highest obtained base-line, [8, 9], and is characterized by perivascular and interstitial mononuclear cell infiltrates or chronic rejection characterized by dense scarring and eosinophilic infiltrates. If rapid deterioration of pulmonary function was detected as a sign of chronic allograft dysfunction, bronchoscopies with TBB was conducted and anti-rejection treatment was initiated with pulsed metylprednisolon often together with tacrolimus or everolimus as a replacement for cyclosporine. In this study, patients with BOS grade ≥ 2 was included and chosen for analysis.

### Statistical methods

The statistical calculations were performed using SPSS Version 19.0. (IBM Corp, Armonk, NY). Primary stratification of the material was made into two sets of cohorts. The first cohort was based on the main indication for LTx, with the following indicator cohorts: COPD, AAT1, CF, PH, and PF. The second set divided the material based on type of LTx: DLTx or SLTx. The aim of this study was to analyze the occurrence of BOS (grade ≥ 2) after primary LTx. In this analysis, death acted as a competing risk event to BOS. In a competing-risks model, we analyzed incidence of BOS grade ≥ 2 and death as two separate outcomes. Specifically, we estimated and compared the cumulative incidence functions for BOS grade ≥ 2 and death using Gray’s test, Gray (1988). All calculations regarding competing risks were performed using R with the CMPRSK package (available at http://www.r-project.org). For all statistical analyses, a *p*-value less than 0. 05 was considered significant. All statistical calculations were performed by Sidesoft AB, Malmo, Sweden.

## Results

### Cumulative incidence of BOS grade ≥ 2 and death

#### Type of transplant

**Fig. 1:**
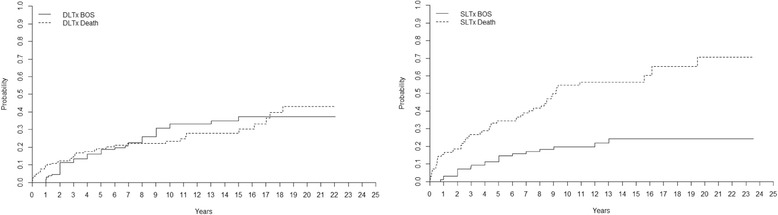
Cumulative incidence of BOS grade ≥ 2 and mortality after LTx in DLTx and SLTx recipients. Note that DLTx and SLTx recipients have the same risk of developing BOS, but DLTx has a significantly better chance of survival despite the presence of BOS

**Fig. 2:**
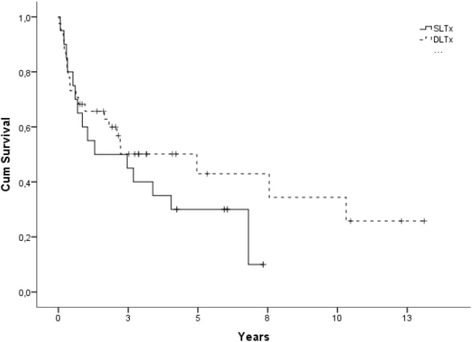
Kaplan-Meier figure displaying survival between SLTx and DLTx after development of BOS grade ≥ 2 until death/follow-up (*p* > 0. 05)

Incidence of BOS (grade ≥ 2) is presented (percentage of probability ± SE) by type of transplant (DLTx and SLTx) in Fig. 1. The incidence of BOS among DLTx-recipients was 16 ± 3% at 5 years, 30 ± 4% at 10 years, 35 ± 5% at 15 years, and 37 ± 5% at 20 years, compared to SLTx-recipients whose incidence of BOS was 11 ± 3% at 5 years, 20 ± 4% at 10 years, 24 ± 5% at 15 years, and 24 ± 5% at 20 years (*p* > 0. 05). The mortality rate for DLTx recipients was 19 ± 3% at 5 years, 23 ± 4% at 10 years, 28 ± 4% at 15 years, and 43 ± 7% at 20-years compared to the mortality rate of SLTx-recipients, which was 34 ± 5% at 5 years, 55 ± 6% at 10 years, 56 ± 6% at 15 years, and 71 ± 8% at 20 years (*p* < 0. 05). Kaplan-Meier survival is displayed after development of BOS (grade ≥ 2) until follow-up/death in Fig. 2. Survival curves are divided into patients that have underwent DLTx vs. SLTx (*p* > 0. 05).

#### Major indications

**Fig. 3:**
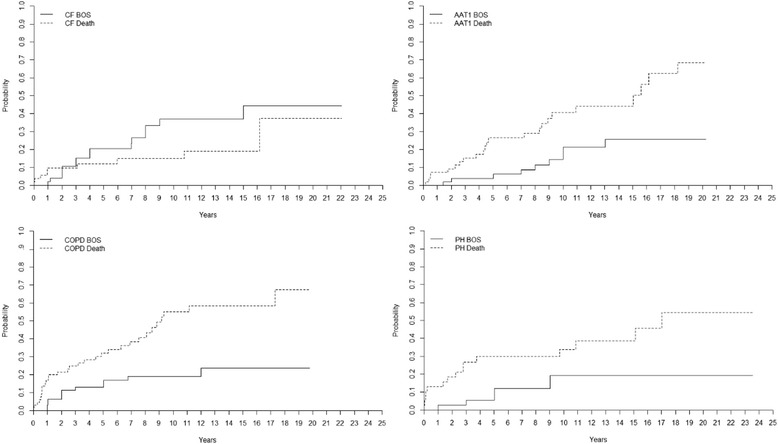
Cumulative incidence of bronchiolitis obliterans syndrome (BOS) and mortality after lung transplantation (LTx) group wise comparing cystic fibrosis (CF), alpha1-antitrypsine deficiency (AAT1) recipients, COPD-recipients and pulmonary hypertension (PH) recipients. CF recipients had a significantly higher risk of developing BOS grade ≥ 2 compared to AAT1 recipients (*p* < 0. 05), but AAT1 had a significantly higher mortality (*p* < 0. 05), indicating that CF recipients might withstand BOS better than AAT1 recipients. Recipients with CF and COPD had the same incidence of BOS grade ≥ 2 (*p* > 0. 05), but chronic obstructive pulmonary disease (COPD) recipients had a significantly higher mortality (*p* < 0. 05), indicating that CF recipients might withstand BOS better than COPD recipients. CF recipients had a significantly higher risk of developing BOS grade ≥ 2 compared to PH recipients. However, CF and PH recipients showed the same mortality, indicating that CF and PH recipients with BOS have the same chance of survival

The incidence of BOS (grade ≥ 2) is presented by different diagnostic groups in Fig. 3. The incidence of BOS among AAT1-patients was 4 ± 3% at 5 years, 14 ± 6% at 10 years, 26 ± 8% at 15 years, and 26 ± 8% at 20 years. For CF-patients it was 20 ± 6% at 5 years, 37 ± 8% at 10 years, 37 ± 8% at 15 years, and 44 ± 10% at 20 years. For COPD-patients it was 13 ± 4% at 5 years, 19 ± 5% at 10 years and 24 ± 7% at 15 years. For PF-patients, the incidence of BOS was 25 ± 8% at 5 years, 34 ± 9% at 10 years, 34 ± 9% at 15 years and 34 ± 9% at 20 years while for PH-patients it was 6 ± 4% at 5 years, 19 ± 7% at 10 years, 19 ± 9% at 15 years, and 19 ± 7% at 20 years (*p* < 0. 05). The mortality rate for AAT1-patients was 26 ± 6% at 5 years, 41 ± 8% at 10 years, 44 ± 8% at 15 years and 68 ± 10% at 20 years. For CF-patients it was 12 ± 5% at 5 years, 15 ± 5% at 10 years, 19 ± 7% at 15 years, and 37 ± 19% at 20 years. For COPD-patients it was 32 ± 6% at 5 years, 55 ± 8% at 10 years, and 58 ± 8% at 15 years. For PF-patients it was 26 ± 8% at five years, 38 ± 9% at 10 years, 38 ± 9% at 15 years, and 52 ± 16% at 20 years, and for PH it was 30 ± 8% at 5 years, 34 ± 8% at 10 years, 39 ± 9% at 15 years, and 54 ± 12 at 20 years (*p* < 0. 05).

#### Major indications compared group wise

The patient groups of major indications were compared. The group “other” describes a heterogenic group of patients who underwent LTx due to bronchiectasis, sarcoidosis, bronchioalveolary cancer, silicosis, BOS and graft-vs-host disease (GVHD). The group had a higher incidence of BOS compared to COPD (*p* = 0. 007), AAT1 (*p* = 0. 001) and PH (*p* = 0. 002) patients. The group also showed significant lower risk of death compared to COPD (*p* = 0. 037), AAT1 (*p* = 0. 281), and PH (*p* = 0. 300) patients.

Recipients with CF had higher risk of developing BOS compared to AAT1 recipients (*p* = 0. 048), but AAT1 had a higher mortality (*p* = 0. 020). Recipients with CF and COPD had the same incidence of developing BOS (*p* = 0. 164), but COPD recipients had a higher mortality (*p* = 0. 001). Recipients with CF had higher risk of developing BOS compared to PH recipients (*p* = 0. 055), but CF and PH recipients had the same mortality (*p* = 0. 057) (Fig. 3).

#### Age and BOS

**Fig. 4:**
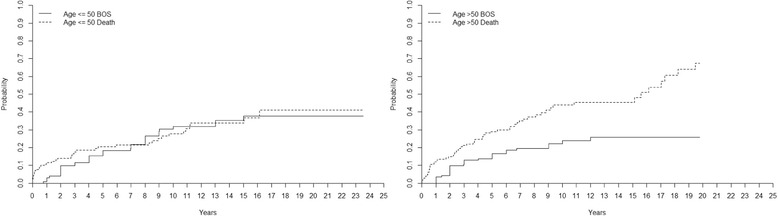
Competing risk analyzing the impact of age on the development of bronchiolitis obliterans syndrome (BOS) and the risk of death after lung transplantation (LTx). Age had no impact on the development of BOS grade ≥ 2, but recipients 50 years or older had a 9% higher mortality 5 years post-transplant and a 16% increased risk 10 years post-transplant compared to recipients younger than 50 years (*p* < 0. 05)

The incidence of BOS (grade ≥ 2) for patients ≤50 years of age was 15 ± 3% at 5 years, 30 ± 5% at 10 years, 35 ± 5% at 15 years, and 38 ± 6% at 20 years. For patients >50 years of age it was 14 ± 3% at 5 years, 22 ± 4% at 10 years, and 26 ± 5% at 15 years (*p* = 0. 238). The mortality rate for patients ≤50 years of age was 20 ± 4% at 5 years, 28 ± 4% at 10 years, 34 ± 5% at 15 years and 41 ± 7% at 20 years. For patients >50 years of age the mortality rate was 29 ± 4% at 5 years, 44 ± 5% at 10 years, and 45 ± 5% at 15 years (*p* = 0. 019) (Fig. 4).

#### Different time periods and BOS

**Fig. 5:**
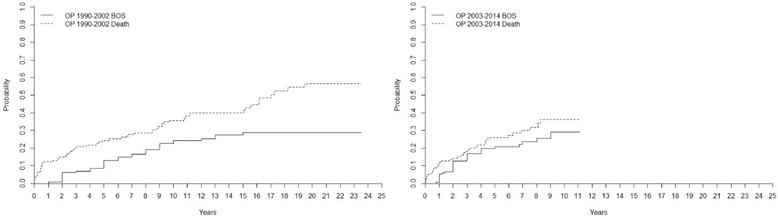
Cumulative incidence of bronchiolitis obliterans syndrome (BOS) and mortality after lung transplantation (LTx) for the two different time periods 1990–2002 and 2003–2014. Our findings (Fig. 1) indicate that DLTx and SLTx carried the same risk of developing BOS grade ≥ 2, but DLTx had a significantly lower risk of death. We suspect that these results might reflect a change in postoperative care towards more aggressive infection and rejection therapy in combination with less frequent SLTx in favor of DLTx the last 10–12 years. However, our results could not confirm these suppositions: no difference was found between the risk of developing BOS grade ≥ 2 or death in different time periods. In Fig. 5 we show the results, supporting the claim that DLTx has a significantly lower risk of death compared to SLTx

For the period 1990–2002, the incidence of BOS (grade ≥ 2) in all recipients was 9 ± 3% at 5 years, 23 ± 4% at 10 years, 27 ± 4% at 15 years, and 29 ± 4 at 20 years. The overall mortality rate for the same time period was 24 ± 4% at 5 years, 36 ± 4% at 10 years, 40 ± 5% at 15 years, and 57 ± 6% at 20 years. Between 2003 and 2014, the incidence of BOS was 8 ± 2% at 2 years, 17 ± 3% at 4 years, 21 ± 4% at 6 years, 24 ± 4% at 8 years, and 29 ± 5% at 10 years. The overall mortality rate for the same time period was 14 ± 3% at 2 years, 22 ± 4% at 4 years, 27 ± 4% at 6 years, 32 ± at 8 years, and 36 ± 5% at 10 years (Fig. 5).

### Mortality

Post-operative cause of death before and after 12 months is shown in Table 2. The group called “other causes” is defined as mortality caused by myocardial and cerebral ischaemia, and multiple organ failure as well as other causes related to the patient’s age and health status.

**Table 2:** Cause of death according to recipient transplantation type and time after transplantation

Tx-type; Cause of Death	< 12 months	> 12 months	*p*-value
Total: 278			
SLTx (*n* = 97)			
Total number of deaths	17	51	0.158
Death from Organ Rejection	2 (12%)	10 (20%)	
Death from Infection	4 (23%)	16 (31%)	
Death from Malignancy	1 (6%)	10 (20%)	
Death from Other Causes	10 (59%)	15 (29%)	
DLTx (*n* = 172)			
Total number of deaths	16	47	0.388
Death from Organ Rejection	4 (25%)	20 (42%)	
Death from Infection	4 (25%)	6 (13%)	
Death from Malignancy	1 (6%)	6 (13%)	
Death from Other Causes	7 (44%)	15 (32%)	
HLTx (n = 9)			
Total number of deaths	1	4	0.576
Death from Organ Rejection	0 (0%)	1 (25%)	
Death from Infection	0 (0%)	0 (0%)	
Death from Malignancy	0 (0%)	0 (0%)	
Death from Other Causes	1 (100%)	3 (75%)	

The group called ‘other causes’ is defined as patients with mortality caused by myocardial and cerebral ischaemia, and multiple organ failure such as renal and liver in addition to other causes related to the patient’s old age and individual health status

## Discussion

Lung transplantation is an established treatment for end stage pulmonary disease [10]. The number of clinical lung transplantation is limited by the shortage of organs, which have resulted in a constant searching for new ways to increase the number of organs [11–14], while survival after lung transplantation mainly limited by CLAD. Survival after lung transplantation has improved significantly over the last decade, however, CLAD, predominantly manifesting as BOS, remains the primary cause of morbidity and mortality after LTx. Although the risk of developing BOS within the first year is low, cumulative incidence of BOS quickly increases within the first five years [9, 15, 16].

The risk factors for BOS are still not fully understood [17]. Anti-human leukocyte antigen (HLA) donor-specific antibodies (DSA) have been associated with early and high-grade BOS and death after LTx in some studies but is still controversial [18, 19]. Treatments to remove antibodies or limit antibody-mediated damage using plasmapheresis have been shown to have some effect when DSA are first detected. However, the impact of this treatment on clinical outcome following LTx remains unclear [20]. Bacterial and viral infection has also been identified as a possible trigger of BOS after LTx [21, 22]. Although BOS was generally thought to be irreversible, recent evidence suggests that some patients with BOS may respond to azithromycin with an improvement of their FEV1 with more than 10% [22]. In addition, another form of chronic rejection, restrictive allograft syndrome (RAS), has recently been described, which does not fit the BOS definition but is instead characterized by restrictive functional changes involving peripheral lung pathology, leading to the introduction of the more encompassing term CLAD [5, 23].

The overall cumulative incidence of BOS grade ≥ 2 among our 278 recipients was 15% after 5 years, 26% after 10-years, 30% after 15-years, and 32% after 20 years post-transplant. The incidence of BOS was highest among the group referred to as “other”. This group describes a heterogenic group of patients who underwent LTx due to bronchiectasis, sarcoidosis, bronchioalveolary cancer, silicosis, BOS, and graft-vs-host disease (GVHD) that might reflect the high incidence of BOS. The group’s heterogenic appearance makes it difficult to draw any conclusions. Besides the group “other,” the highest incidence of BOS was seen among PF recipients followed by CF, COPD, PH, and AAT1 recipients in the described descending order. It has been shown that BOS and PF respectively exhibit similar disease characteristics with overlapping pathophysiology such as epithelial cell injury and increase in production/deposition of ECM [24]. This patient group is of great interest as the identification of biomarkers in PF could contribute to finding new means of earlier finding BOS [25]. The highest mortality was seen among COPD recipients followed by PH, AAT1, PF, and CF recipients in the described descending order, indicating that CF and PF recipients have a better chance of survival despite developing BOS compared to the other major indications such as COPD and PH. However, it should also be acknowledged that patient outcome among LTx-recipients in Sweden might differ in comparison to other countries such as the US due to a significantly higher incidence of COPD/CF vs. interstitial lung disease, in combination with Sweden having younger recipients [26]. As well as reports from the ISHLT showing almost double the incidence of interstitial lung disease in LTx in comparison to Sweden.

When we compared the different patient groups (pairs of two) of major indications between each other, the group referred to as “other” had significantly higher risk of developing BOS (grade ≥ 2) compared to COPD, AAT1, and PH recipients, keeping in mind that this group of recipients reflects a heterogeneous group of patients where some of the recipients underwent LTx due to BOS and GVHD and where the recipients probably already at the time of transplantation have an immunologic response that might lead to the development of BOS in the pulmonary graft.

Recipients with CF had a significantly higher risk of developing BOS compared to AAT1 recipients, but AAT1 had a significantly higher mortality rate, indicating that CF recipients might withstand the development of BOS better than AAT1 recipients. This finding could have a positive effect on the clinical implication of favoring CF recipients in LTx. Previously the overall survival benefit of LTx in CF has been reported as controversial due to associated risk factors such as CF-related arthropathy as well as associated chronic infections with bacterial/fungal agents like *B.cepacia*, *P.Auriginosa* and *Aspergillus* that can be serious and life-threatening [27]. Interestingly, recipients with CF had a significantly higher risk of developing BOS compared to PH recipients, however, CF and PH recipients had the same mortality, indicating that CF and PH recipients developing BOS have the same chance of survival despite BOS. It has been shown that PH patients undergo extensive remodeling of the pulmonary arterial walls as part of their pathophysiology, leading to permanent changes of the intima [28]. However, PH is also seen post-LTx among different recipients as bronchiolitis obliterans is often associated with immune-mediated arterio- and venopathy leading to pre- and post-capillary PH [29]. It could be theorized that patients with PH prior to LTx might better withstand this phenomenon, indicating why PH recipients don’t show inferior survival to CF despite BOS. The clinical implication of understanding the development of BOS in this disease state could be of immense potential. This hypothesis was however not investigated in this study with the need of further data.

Recipients with CF and COPD had the same incidence of BOS, but COPD recipients had a significantly higher mortality, indicating that CF recipients might withstand the development of BOS better than COPD recipients. COPD recipients are often older than CF patients at the time of transplant, and the COPD patients often have comorbidities such as heart and vascular disease that might in part explain these results. We did an expanded analysis to investigate the impact of age on the development of BOS and the risk of death. We concluded that age had no impact on the development of BOS, but recipients 50 years or older had a 9% higher mortality 5 years post-transplant and a 16% increased risk after 10 years post-transplant compared to recipients younger than 50 years (*p* < 0. 05).

Interestingly, when we compared DLTx to SLTx in the entire cohort we found that DLTx and SLTx recipients had equal risk of developing BOS grade ≥ 2. However, recipients receiving DLTx had a significantly better chance of surviving. These results further support a clinical program favoring DLTx instead of SLTx. We tried to analyze the pattern of DLTx and SLTx among major indications such as COPD, but unfortunately the groups were not big enough to reach statistical evaluation. Our findings indicate that DLTx and SLTx had the same risk of developing BOS, but DLTx had a better chance of survival. We suspect that these results might reflect the fact that during the last 10–15 years, we have initiated early treatment for viral and bacterial infection, used more aggressive therapy at the first sign of rejection, and favored DLTx over SLTx. The frequency of SLTx reached its peak in 2002 at our clinic and has significantly declined since then in favor of DLTx. However, our results did not support these hypotheses as no difference was found between the risk of BOS or mortality in different time periods. In Fig. 5 we show the results for the two different time periods 1990–2002 and 2003–2014.

### Limitations

There have been significant changes in the care of transplant patients over the last 25 years, which affects outcome variables such as survival depending on year of transplantation. Recipient inclusion criteria have broadened over the years and now preoperative ECMO support or ventilator support are no longer contraindications for LTx, representing a complex recipient clientele. Several confounding factors have been linked to long-term survival in LTx that could be playing a role here. Such variables are recipient/donor age and total lung capacity in addition to recipient kidney function, O2 requirement and allograft ischemic time. In the present study patients diagnosed as BOS grade 1 did not, in the majority, of the cases get a specific treatment or an alternation in the present regime even though the patient was diagnosed with BOS. It is possible to diagnose BOS through spirometry, where according to ISHLT a drop in FEV1 more than 20% from baseline is associated with BOS grade ≥ 1. Though possible confounders that might affect post-operative pulmonary function besides BOS are recurring infections or a decline in FEV1 affected by the natural aging-process or other comorbidities. It should also be acknowledged that the definition of FEV1 < 80% from best baseline for BOS is debatable in comparison to current CLAD criteria, such as phenotyping into BOS vs. RAS. To differentiate those who really have a rejection (BOS) or not, we have in this analysis calculated BOS as BOS grade 2 or more. This can of course be cautious to follow.

## Conclusions

No differences were seen in the incidence of BOS grade ≥ 2 regarding type of transplant, however, DLTx recipients showed a better chance of survival despite the same risk for developing BOS compared to SLTx recipients, indicating that recipients receiving DLTx withstand BOS better than SLTx. These figures further support a clinical program favoring DLTx instead of SLTx. The highest incidence of BOS grade ≥ 2 was seen among PF, CF, COPD, PH, and AAT1 recipients in descending order described where PF recipients had the highest risk of developing BOS. However, CF and PF recipients showed a better chance of survival despite developing BOS compared to COPD, PH, and AAT1 recipients.

## References

[1] Verleden SE, Vasilescu DM, Willems S (2014). The site and nature of airway obstruction after lung transplantation. Am J Respir Crit Care Med.

[2] Gauthier JM, Hachem RR, Kreisel D (2016). Update on chronic lung allograft dysfunction. Curr Transplant Rep.

[3] Al-Githmi I, Batawil N, Shigemura N (2006). Bronchiolitis obliterans following lung transplantation. Eur J Cardiothorac Surg.

[4] Vandermeulen E, Lammertyn E, Verleden SE (2017). Immunological diversity in phenotypes of chronic lung allograft dysfunction: a comprehensive immunohistochemical analysis. Transpl Int.

[5] Verleden SE, Vanaudenaerde BM, Vos R, Verleden GM (2016). Phenotypes of chronic lung allograft dysfunction: getting closer step by step?. Am J Transplant.

[6] Verleden SE, Sacreas A, Vos R (2016). Advances in understanding bronchiolitis obliterans after lung transplantation. Chest.

[7] Royer PJ, Olivera-Botello G, Koutsokera A (2016). Chronic lung allograft dysfunction: a systematic review of mechanisms. Transplantation.

[8] Estenne M, Maurer JR, Boehler A (2002). Bronchiolitis obliterans syndrome 2001: an update of the diagnostic criteria. J Heart Lung Transplant.

[9] Verleden GM, Raghu G, Meyer KC (2014). A new classification system for chronic lung allograft dysfunction. J Heart Lung Transplant.

[10] Fakhro M, Ingemansson R, Skog I (2016). 25-year follow-up after lung transplantation at Lund University Hospital in Sweden: superior results obtained for patients with cystic fibrosis. Interact Cardiovasc Thorac Surg.

[11] Lindstedt S, Pierre L, Ingemansson R (2013). A short period of ventilation without perfusion seems to reduce atelectasis without harming the lungs during ex vivo lung perfusion. J Transp Secur.

[12] Lindstedt S, Eyjolfsson A, Koul B (2011). How to recondition ex vivo initially rejected donor lungs for clinical transplantation; clinical experience from Lund University Hospital. J Transp Secur.

[13] Machuca TN, Cypel M (2014). Ex vivo lung perfusion. J Thorac Dis.

[14] Sanchez PG, Bittle GJ, Williams K (2013). Ex vivo lung evaluation of prearrest heparinization in donation after cardiac death. Ann Surg.

[15] Christie JD, Edwards LB, Kucheryavaya AY (2011). The registry of the International Society for Heart and Lung Transplantation: twenty-eighth adult lung and heart-lung transplant report--2011. J Heart Lung Transplant.

[16] Traxler D, Schweiger T, Schwarz S (2017). The lymphatic phenotype of lung allografts in patients with bronchiolitis obliterans syndrome and restrictive allograft syndrome. Transplantation.

[17] Olland A, Reeb J, Leclerq A (2016). Microparticles: a new insight into lung primary graft dysfunction?. Hum Immunol.

[18] Westall GP, Paraskeva MA, Snell GI. Antibody-mediated rejection. Curr Opin Organ Transplant. 2015; 10.1097/MOT.0000000000000235 26262460

[19] Zazueta OE, Preston SE, Moniodis A, et al. The presence of Pretransplant HLA antibodies does not impact the development of chronic lung allograft dysfunction or CLAD related death. Transplantation. 2016; 10.1097/TP.0000000000001494 27893614

[20] Safavi S, Robinson DR, Soresi S (2014). De novo donor HLA-specific antibodies predict development of bronchiolitis obliterans syndrome after lung transplantation. J Heart Lung Transplant.

[21] Luckraz H, Sharples L, McNeil K (2003). Cytomegalovirus antibody status of donor/recipient does not influence the incidence of bronchiolitis obliterans syndrome in lung transplantation. J Heart Lung Transplant.

[22] Vos R, Vanaudenaerde BM, Ottevaere A (2010). Long-term azithromycin therapy for bronchiolitis obliterans syndrome: divide and conquer?. J Heart Lung Transplant.

[23] Verleden SE, Ruttens D, Vandermeulen E (2015). Restrictive chronic lung allograft dysfunction: where are we now?. J Heart Lung Transplant.

[24] Fernandez IE, Eickelberg O (2012). New cellular and molecular mechanisms of lung injury and fibrosis in idiopathic pulmonary fibrosis. Lancet.

[25] Heijink IH, Rozeveld D, van der Heide S (2015). Metalloproteinase profiling in lung transplant recipients with good outcome and bronchiolitis obliterans syndrome. Transplantation.

[26] Kistler KD, Nalysnyk L, Rotella P, Esser D (2014). Lung transplantation in idiopathic pulmonary fibrosis: a systematic review of the literature. BMC Pulm Med.

[27] Lobo LJ, Noone PG (2014). Respiratory infections in patients with cystic fibrosis undergoing lung transplantation. Lancet Respir Med.

[28] Wilkinson M, Langhorne CA, Heath D (1988). A pathophysiological study of 10 cases of hypoxic cor pulmonale. Q J Med.

[29] Saggar R, Ross DJ, Saggar R (2008). Pulmonary hypertension associated with lung transplantation obliterative bronchiolitis and vascular remodeling of the allograft. Am J Transplant.

